# Genotype Distribution, Viral Load and Clinical Characteristics of Infants with Postnatal or Congenital Cytomegalovirus Infection

**DOI:** 10.1371/journal.pone.0108018

**Published:** 2014-09-30

**Authors:** Joppe Nijman, Femke S. Mandemaker, Malgorzata A. Verboon-Maciolek, Susan C. Aitken, Anton M. van Loon, Linda S. de Vries, Rob Schuurman

**Affiliations:** 1 Department of Virology, University Medical Center, Utrecht, The Netherlands; 2 Department of Neonatology, University Medical Center, Utrecht, The Netherlands; University of British Columbia, Canada

## Abstract

**Background:**

Congenital cytomegalovirus infection is a leading cause of long-term sequelae. Cytomegalovirus is also frequently transmitted to preterm infants postnatally, but these infections are mostly asymptomatic. A correlation between cytomegalovirus genotypes and clinical manifestations has been reported previously in infants with congenital infection, but not in preterm infants with postnatal infection.

**Objectives:**

The main objective of this study was to investigate cytomegalovirus genotype distribution in postnatal and congenital cytomegalovirus infection and its association with disease severity.

**Methods:**

Infants admitted to the neonatal intensive care unit of the University Medical Center Utrecht, The Netherlands between 2003–2010 and diagnosed with postnatal or congenital cytomegalovirus infection were included. Classification of cytomegalovirus isolates in genotypes was performed upon amplification and sequencing of the cytomegalovirus UL55 (gB) and UL144 genes. Clinical data, cerebral abnormalities, neurodevelopmental outcome and viral load were studied in relation to genotype distribution.

**Results:**

Genotyping results were obtained from 58 preterm infants with postnatal cytomegalovirus infection and 13 infants with congenital cytomegalovirus infection. Postnatal disease was mild in all preterm infants and all had favourable outcome. Infants with congenital infection were significantly more severely affected than infants with postnatal infection. Seventy-seven percent of these infants were symptomatic at birth, 2/13 died and 3/13 developed long-term sequelae (median follow-up 6 (range 2–8) years). The distribution of cytomegalovirus genotypes was comparable for postnatal and congenital infection. UL55 genotype 1 and UL144 genotype 3 were predominant genotypes in both groups.

**Conclusions:**

Distribution of UL55 and UL144 genotypes was similar in asymptomatic postnatal and severe congenital CMV infection suggesting that other factors rather than cytomegalovirus UL55 and UL144 genotype are responsible for the development of severe disease.

## Introduction

Cytomegalovirus (CMV) infection is the most common congenital infection. Symptoms of CMV infection at birth include intrauterine growth restriction, microcephaly, petechiae, jaundice, hepatomegaly, retinitis, hearing loss, cerebral abnormalities and thrombocytopenia. Congenital CMV infection may lead to death or neurologic sequelae, including sensorineural hearing loss later in life [1], [2].

CMV is also transmitted postnatally to newborn infants, mostly via breast milk of their CMV seropositive mothers [3]. Term born infants have an insignificant risk of symptomatic postnatal CMV infection, which is related to the transplacental transfer of anti-CMV antibodies [4]. In contrast, symptoms of postnatal CMV infection in preterm (gestational age <32 weeks) or very low birth weight (<1500 grams) infants may be severe and even life-threatening and may include sepsis-like illness, pneumonia and thrombocytopenia. However, the majority of the postnatally infected preterm infants are also asymptomatic [5]. Limited data suggests that neurodevelopmental outcome and hearing are favourable in these preterm infants [6]–[8].

It has been hypothesized that differences between CMV strains may contribute to differences in virus-associated clinical manifestations [9]. In various studies, considerable genetic variability was found in CMV UL55 (gB) and UL144, enabling the assignment of specific CMV genotypes for each of both genes. UL55 or UL144 based genotyping has been applied to investigate either epidemiological or clinical associations of (congenital) CMV infection in term infants [10]–[17]. Correlations between CMV genotypes and clinical manifestations are still limited and controversial, potentially due to the heterogeneous composition of the investigated cohorts.

The aim of the present study was to determine whether postnatal and congenital CMV infections in (pre-)term infants were caused by specific CMV UL55 and UL144 genotypes and to assess whether these genotypes were associated with CMV disease severity. In addition, the correlation between genotype distribution and viral load was evaluated.

## Materials and Methods

### Study population

Newborn infants admitted to the Neonatal Intensive Care Unit of the University Medical Center, Utrecht, The Netherlands, and diagnosed with postnatal or congenital CMV infection between 2003–2010 were included. CMV diagnostics were performed in term infants with symptoms of congenital CMV infection (between 2003–2010), in term infants with proven maternal seroconversion (between 2003–2010) or in preterm infants as part of a screening study on postnatal CMV infection (between 2007–2010) [5].

Postnatal CMV infection in preterm infants was determined when the CMV PCR in urine collected at term-equivalent age (40 weeks postmenstrual age) was positive, while the urine collected within two weeks after birth was CMV negative. In infants of whom urine was not collected after birth, a negative CMV PCR on dried blood spots cards obtained within 3 days after birth was used to exclude congenital CMV infection, as described previously [18].

Congenital CMV infection was diagnosed if a CMV PCR in urine was positive within 2 weeks after birth. Symptomatic postnatal CMV disease was diagnosed in infants with clinical manifestations of CMV disease during admission (sepsis-like illness, pneumonia or thrombocytopenia [<150×10∧9/L]), negative bacterial blood cultures and positive CMV PCR in urine and blood at time of symptoms.

Demographic and clinical data and CMV urine loads were collected, as defined earlier [5]. Cerebral abnormalities were evaluated using cranial ultrasonography and magnetic resonance imaging (MRI) during admission. Cranial ultrasonography was repeated every week by experienced neonatologists in preterm infants during their stay in the Neonatal Intensive Care Unit. The MRI was performed in term infants with congenital CMV infection shortly after birth and in preterm infants with congenital or postnatal CMV infection at term-equivalent age using a 3-Tesla Intera system or a 1.5-Tesla system (Philips, The Netherlands). The results of the cranial ultrasonography and MRI were retrospectively reviewed by two neonatologists with>20 years of experience in neonatal neuro-imaging, as reported previously [5], [19]. Brainstem auditory evoked potentials were performed to determine sensorineural hearing loss.

Sample collection was part of standard patient care and therefore, informed consent was not obtained. Oral and written information about taking a urine sample was given to the parents. In case of a positive CMV PCR, parents were informed and additional hearing assessment was performed over the ensuing years. All data for this study were used and analyzed in strictly anonymous form, according to the code of conduct for medical research approved by the hospital's Medical Ethical Committee. The Medical Ethical Committee (Medisch Ethische Toetsingscommissie) of the hospital approved the consent procedure and current study.

### DNA extraction, Polymerase Chain Reaction and sequencing

Viral DNA was isolated from 100 µl urine using the Nuclisens MiniMAG (BioMérieux, Boxtel, The Netherlands) and eluted in 100 µl elution buffer. The purified DNA was subsequently used for amplification of the UL55 and UL144 genes, using both sets of outer primers described in Table 1, which are essentially based on Lurain et al. [10] and Stranska et al. [11] (Life Technologies, Foster City, CA, USA). A 616-bp fragment containing the UL144 coding sequence and a 532-bp fragment containing the UL55 coding sequence were amplified using the Expand-high fidelity amplification kit (Roche Applied Science, Penzberg, Germany). PCR was performed in a GeneAmp2700 thermal cycler (Life technologies), according to the following conditions: 1 hold at 94°C for 2 minutes, 10 cycles at 94°C for 15 seconds, 55°C for 30 seconds and 72°C for 1 minute, 20 cycles at 94°C for 15 seconds, 55°C for 30 seconds and 72°C for 1 minute and 5 seconds (increased by 5 seconds every cycle), final extension at 72°C for 7 minutes. Nested PCR was performed according to the following conditions: 1 hold at 94°C for 2 minutes, 10 cycles at 94°C for 15 seconds, 55°C for 30 seconds and 72°C for 45 seconds, 10 cycles at 94°C for 15 seconds, 55°C for 30 seconds and 72°C for 50 seconds (increased by 5 seconds every cycle), final extension at 72°C for 7 minutes.

**Table 1:** Sequences of oligonucleotide primers used for CMV PCR and sequencing (12,27).

Primer name	Sequence
*Primers used for amplification*	
UL55_Outer_Forward	5′-TCCGAAGCCGAAGACTCGTA-3′
UL55_Outer_Reverse	5′-CATTCCTCAGTGCGGTGGTT-3′
UL55_Inner_Forward	5′-CTGCCAAAATGACTGCAACT-3′
UL55_Inner_Reverse	5′-ACATCACCCATGAAACGCGC-3′
UL144_Outer_Forward	5′-ACAAACCGCGGAGAGGATGA-3′
UL144_Outer_Reverse	5′-TCAGACACGGTTCCGTAAAG-3′
UL144_Inner_Forward	5′-GTTCGGCCCCATGAGTTATT-3′
UL144_Inner_Reverse	5′-GTGTGACTTCATCGTACCGT-3′
*Primers used for sequencing*	
UL55_Sequencing_Forward	5′-CTGCCAAAATGACTGCAACT-3′
UL55_Sequencing_Reverse	5′-ACATCACCCATGAAACGCGC-3′
UL144_Sequencing_Forward	5′-GTTCGGCCCCATGAGTTATT-3′
UL144_Sequencing_Reverse	5′-GTGTGACTTCATCGTACCGT-3′

PCR-products were visualized upon agarose gel electrophoresis, purified using QIAquick PCR purification columns (Qiagen Gmbh, Germany) following the manufacturer's instructions and eluted in 30–100 µl nuclease-free water.

The purified PCR-products were subsequently used for cycle sequencing in a GeneAmp2700 thermal cycler, according to the following protocol: 25 cycles at 96°C for 10 seconds, 50°C for 5 seconds and 60°C for 4 minutes.

Reaction products were purified using EDTA-ethanol precipitation, prior to analysis on an ABI3700 automated sequencer (Life technologies), using the primers described in Table 1.

The obtained electropherograms were analyzed and edited using SeqScape data analysis software, v2.6 (Life technologies). Full double stranded coverage was achieved for both amplified gene fragments. The obtained consensus sequences for each of the samples were then aligned with MEGA Alignment Explorer and genotype classification was achieved with MEGA Tree Explorer (MEGA, The Biodesign Institute, AZ, USA). UL144 sequences were classified into genotype 1A, 1B, 1C, 2 or 3, according to Lurain et al. [10], which essentially correspond to genotype A, A/C, A/B, C or B, respectively, according to the classification of Arav-Boger et al. [16] UL55 sequences were classified into genotype 1, 2, 3, 4 or 5, based on several studies [10], [12], [16], [20]–[23]. The reference sequences used for classification were obtained from GenBank, Pubmed.

### Statistical analysis

Statistical analysis was performed in PASW statistics (version 18.0, SPSS Inc., 2009). Proportional variables were analyzed using chi-square test or Fisher's exact test. Continuous variables were analyzed using a non-parametric Mann-Whitney U test. To compare CMV load between postnatally and congenitally infected infants, logarithmic transformation was performed. Variances of CMV load between genotypes were analyzed with the non-parametric Kruskal-Wallis test. A p-value <0.05 was considered statistically significant.

## Results

### Clinical data

Clinical data of both groups of patients are summarized in Table 2. The majority of the studied infants were preterm (62/71, 87%) and were identified by means of a previous study on CMV infection in preterm infants [5]. Of 58 preterm infants with postnatal CMV infection, five (9%) developed symptoms of CMV disease (pneumonia [n = 3], sepsis-like illness with thrombocytopenia [n = 2]) while the others (91%) were asymptomatic and identified by a screening program [5]. None of the infants with postnatal CMV infection were treated with antiviral medication.

**Table 2:** Demographic and clinical characteristics of 58 postnatally and 13 congenitally infected infants.

	Postnatal CMV infection	Congenital CMV infection	P
	n = 58	n = 13	
Gestational age, mean, wk (SD)	28.4 (1.9)	36.5 (4.2)	<0.001
Birth weight, mean, g (SD)	1140 (330)	2530 (1030)	<0.001
Male gender, n (%)	33 (57)	8 (62)	0.759
Non-Dutch maternal origin, n (%)	27 (47)	3 (23)	0.121
Symptomatic CMV infection, n (%) ^a^	5 (9)	10 (77)	<0.001
Mortality, n (%)	0 (0)	2 (15)	0.002
Abnormal outcome/hearing, n (%)	0 (0)	3 (23)	<0.001
Urine Log_10_ CMV load, median, copies/mL (IQR)	5.24 (1.43)	6.34 (1.62)	0.002
Severe MRI abnormalities, n (%) ^b^	0 (0)	6 (86)	<0.001
Calcifications on cranial ultrasonography at term-equivalent age, n (%)	20 (35)	6 (46)	0.429

a. Symptoms of postnatal CMV infection included pneumonia (n = 3), and sepsis-like illness with thrombocytopenia (n = 2).

Symptoms of congenital CMV infection included intra-uterine growth retardation (n = 5), microcephaly (n = 2), hepatosplenomegaly (n = 4), petechiae (n = 3), jaundice (n = 1), seizures (n = 1), thrombocytopenia (n = 6), anaemia (n = 2), and neutropenia (n = 1).

b. MRI was performed in 30 postnatally infected infants and 7 congenitally infected infants. Severe MRI abnormalities included polymicrogyria (n = 1), occipital cysts (n = 1), ventricular dilatation (n = 1) and abnormal white matter signal intensity (n = 4).

Infants with congenital CMV infection (n = 13) were affected more severely than infants with postnatal CMV infection. Ten (77%) presented at birth with symptoms of CMV disease: Intrauterine growth restriction (n = 5), microcephaly (n = 2), hepatosplenomegaly (n = 4), petechiae (n = 3), jaundice (n = 1), seizures detected by electroencephalography (n = 1), thrombocytopenia (n = 6), anaemia (n = 2), neutropenia (n = 1). Cranial ultrasonography was performed in all infants and showed ventricular dilatation in two infants and presence of lenticulostriate vasculopathy in six (46%) and germinolytic cysts in seven (54%) infants. In addition, cerebral MRI was performed in seven symptomatic infants and showed severe abnormalities in six infants, including polymicrogyria (n = 1), occipital cysts (n = 1), ventricular dilatation (n = 1) and abnormal white matter signal intensity (n = 4). Two infants died shortly after birth and three developed neurodevelopmental delay or epilepsy and sensorineural hearing loss at 8, 6 and 2 years, respectively. The other eight infants did not develop sequelae, including sensorineural hearing loss (median time of follow-up 1.8 years, range 1–3 years). Two (15%) infants with severe symptoms of congenital CMV infection (seizures [n = 1], cerebral abnormalities [n = 2], hepatosplenomegaly [n = 1] and abnormal automated auditory brainstem response test [n = 2]) were treated with antiviral medication, of whom one developed neurodevelopmental delay and sensorineural hearing loss and one had a favourable outcome. Three asymptomatic infants with congenital infection were born from mothers with proven primary CMV infection during late pregnancy and had normal outcome.

In contrast to infants with congenital infection, none of the infants with postnatal infection developed severe cerebral abnormalities. The presence of lenticulostriate vasculopathy and germinolytic cysts was seen on cranial ultrasonography in 20 (35%) and 8 (14%) infants, respectively, and was not associated with unfavourable neurodevelopmental outcome. No additional cerebral abnormalities were found using MRI, which was performed in 30 (52%) preterm infants with postnatal CMV infection. All infants with postnatal CMV infection had a favourable outcome and normal hearing, determined between 16 months and eight years of life. None of the infants with congenital or postnatal CMV infection developed CMV chorioretinitis.

### Genotype assignment

A total of 86 urine samples from 73 infants were included in the genotypic analysis. Genotyping was successful for both genes in 69 out of 73 (95%) infants, of whom 56 (81%) were postnatally infected and 13 (19%) congenitally infected. In addition, in two infants with postnatal CMV infection only UL144 or UL55 could be genotyped. No genotype could be assigned for 2/73 infants due to a repeatedly negative amplification result. In 13/73 (18%) infants, genotyping was performed on two sequential samples collected between one day and two months after the initial sample. In all cases genotype assignments were identical in both samples (data not shown). Based on the results of population sequencing of the clinical samples, no indications for the presence of mixed genotypes in any of the infants were observed in the electropherograms.

The genotype distribution for UL55 and UL144 was similar for postnatal and congenital CMV infection (Table 3). With 46% CMV UL55 genotype 1 and UL144 genotype 3 were the most prevalent genotypes. There were no differences in UL55 and UL144 genotypes with respect to development of lenticulostriate vasculopathy in both groups (data not shown).

**Table 3:** UL55 and UL144 genotype distribution of postnatal and congenital CMV infection.

Genotype UL55	Postnatal (n = 57) ^a^	Congenital (n = 13)	Total (n = 70)
	n (%)	n (%)	n (%)
1	26 (46)	6 (46)	32 (46)
2	12 (21)	2 (15)	14 (20)
3	11 (19)	3 (23)	14 (20)
4	3 (5)	2 (15)	5 (7)
5	5 (9)	0 (0)	5 (7)
**Genotype UL144**			
1A	10 (18)	4 (31)	14 (20)
1B	2 (4)	1 (8)	3 (4)
1C	1 (2)	0 (0)	1 (1)
2	19 (33)	3 (23)	22 (31)
3	25 (44)	5 (39)	30 (43)

a. In two postnatally infected infants only UL144 or UL55 could be genotyped.

### Viral load

**Figure 1:**
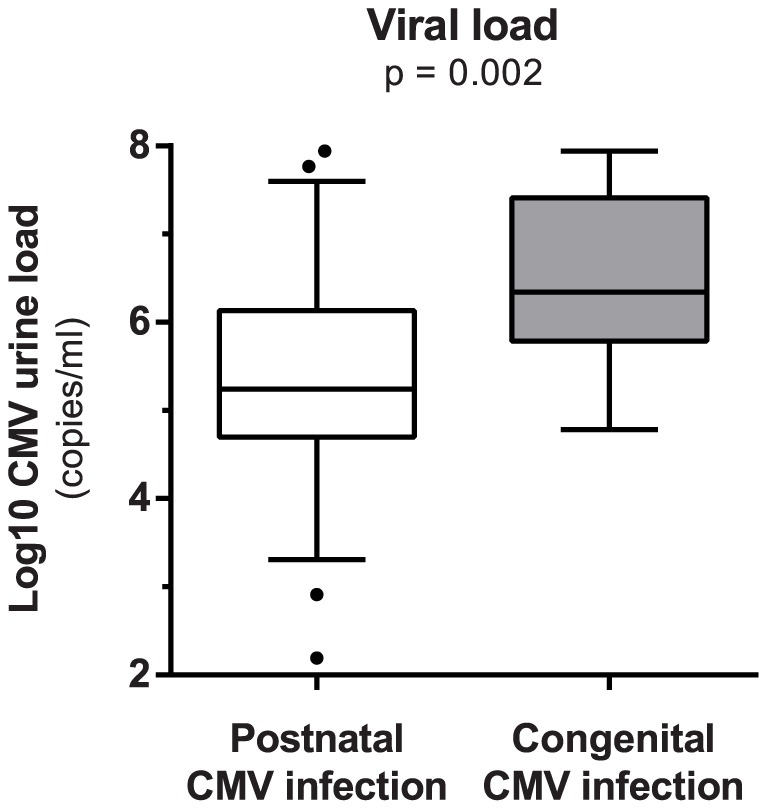
Log_10_ CMV urine load in postnatally and congenitally infected infants. Bar in boxplot represent median viral load after log_10_ transformation. Upper and lower limit of boxplot represent 75^th^ and 25^th^ percentile, respectively. Whiskers represent 5–95% coincidence interval. Dots represent outliers.

CMV load was determined in urine samples of all infants. The median viral load was significantly higher in congenital infection (6.34 Log10 copies/ml [IQR 1.62]) compared to postnatal infection (5.24 Log10 copies/ml [IQR 1.43]), p = 0.002 (Figure 1).

**Figure 2:**
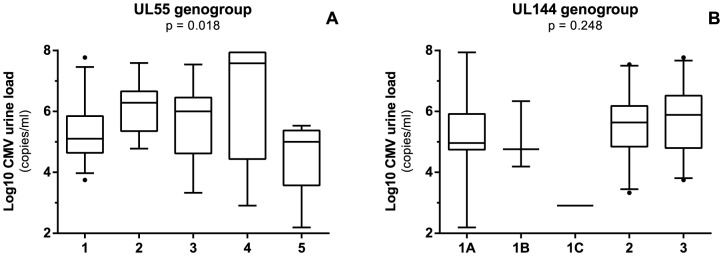
Log_10_ CMV urine load in both postnatally and congenitally infected infants with respect to UL55 genotypes (A) and UL144 genotypes (B). Bar in boxplot represents median viral load after log_10_ transformation. Upper and lower limit of boxplot represent 75^th^ and 25^th^ percentile, respectively. Whiskers represent 5–95% coincidence interval. Dots represent outliers.

In postnatal or congenital CMV infection, there were no statistically significant associations between viral load and UL55 or UL144 genotypes (data not shown). When both patient groups were combined, there was a statistical significant association between viral load and UL55 genotypes (p = 0.018), as shown in Figure 2A. There were no statistical differences in viral load between UL144 genotypes, as shown in Figure 2B.

## Discussion

To date, this is the first study that reported the prevalence of various genotypes in preterm infants with proven postnatal CMV infection, and examined the relationship between genotype and disease severity in both postnatal and congenital CMV infection. UL55 and UL144 genes have been demonstrated to be good candidates for genotyping, because of the significant genetic variation observed in these genes [10], [21].

The distribution of UL55 and UL144 genotypes among the infants with congenital infection in the present study is generally consistent with previously reported data [10], [12], [17], [24]–[26]. UL144 genotype 3 is the predominant genotype (43% of all infants), but UL144 genotype 2 is much more represented in the present population than in the cohort of Lurain et al. [10], 31.4% versus 9.0%, respectively. In accordance with other studies [12], [26]–[28], UL55 genotype 1 was the predominant genotype (46% of all infants). UL155 genotype 2 was reported to be the predominant genotype in Polish term newborns with congenital or postnatal CMV infection (96–100%) [17]. There were no indications for the presence of multiple genotypes in any of the clinical samples. It is to be noted though, that the genotyping procedure was based on bulk sequencing of the amplified genes, which is prone to detect the major genotype. Mixed genotypes would only be detected if the contribution of the minority genotype was at least 25% of the total viral population.

In 2/73 patients the UL55 and UL144 genotypes could be determined for neither of both genes. In each of these cases this was due to a negative PCR result upon amplification of the gene. Despite the well detectable viral loads (>>3.000 copies/ml), the PCR result remained negative for each of the samples upon experiment repeat, which may indicate that the CMV strains of these patients could harbor a mutation in one of the amplification primers. Both of the failing samples were from patients with a postnatal CMV infection. The missing results do not change the overall conclusion of the research regarding the widespread distribution of CMV genotypes in our cohort of congenital or postnatal CMV infections.

This study shows that the UL55 and UL144 genotype distribution of CMV appears similar whether they are vertically (intrauterine) or horizontally (most probably through breast milk) transmitted from mother to infant. Furthermore, we found that the same CMV genotypes are able to cause severe CMV disease, sensorineural hearing loss and neurodevelopmental delay in infants infected congenitally and asymptomatic disease and normal outcome in postnatally infected infants. This may suggest that other factors, including other CMV genotypes, but especially time of onset of the infection [29] and hence, stage of brain maturation, are more important than CMV UL55 or UL144 genotype with respect to development of severe disease.

UL55 genotype 3 was recently found to be associated with severe manifestations of CMV disease in infants with congenital CMV infection [12]. Furthermore, in a study by Yu et al. [24], infants with UL55 genotype 1 and symptomatic CMV disease were diagnosed with CMV related liver disease more frequently. Two other recent studies by Paradowska et al. [30], [31] suggest that certain other CMV genotypes (UL73/gN and UL75/gH, respectively) may predict neurological dysfunction (gN4 genotype) or hearing loss and higher viral load (gH1 genotype) in congenitally and postnatally or unproven congenitally infected infants. Several studies on congenital CMV infection have suggested that blood and urine CMV loads may be important markers of CMV disease severity [24], [25], [31]–[34]. It was recently documented that infants with postnatal CMV infection have significantly lower urine CMV load than infants with congenital CMV infection [35]. In the present study, it is additionally shown that this difference consisted irrespective of genotype distribution. None of these previously reported associations between genotypes, high viral load and development of sequelae could be confirmed in present study.However, this study is limited by the small sample size and selection bias of infants with congenital infection.

With regard of the cranial ultrasonography and MRI results, lenticulostriate vasculopathy and germinolytic cysts were frequently detected in both postnatally and congenitally infected infants (35% and 8% versus 46% and 54%, respectively). Data on the exact incidence of these abnormalities in the CMV negative population are limited, but a prevalence of 4% was reported in a NICU population for LSV and none of these infants had a CMV infection [36]. The incidence for cysts in the absence of underlying etiologies was only 0.5% [37]. Both abnormalities have been previously associated with postnatal and congenital CMV infection, as well as other clinical conditions [5], [38]. The severe neuro-imaging findings in congenitally infected infants correspond to previously reported findings [19].

The sensitivity of CMV PCR of dried blood spots to exclude congenital CMV infection has been discussed. The CMV PCR in the current study is similar to a high-sensitivity method by Leruez-Ville et al. [39] and has been combined with anti-CMV IgM analysis of the dried blood spots, clinical data and viral load to minimize the possibility of misclassification of congenitally infected infants as postnatally infected.

In conclusion, the CMV genotype distribution in asymptomatic postnatally infected preterm infants and severe congenitally infected term infants was similar, which suggests that other factors than CMV UL55 or UL144 genotype may be responsible for the development of disease symptoms and adverse outcome. The role of viral load in relation to genotype needs further study.

## References

[1] Dollard SC, Grosse SD, Ross DS (2007) New estimates of the prevalence of neurological and sensory sequelae and mortality associated with congenital cytomegalovirus infection. Rev Med Virol 17: 355–363 10.1002/rmv 17542052

[2] De Vries JJ, Korver AM, Verkerk PH, Rusman L, Claas EC, et al (2011) Congenital cytomegalovirus infection in the Netherlands: birth prevalence and risk factors. J Med Virol 83: 1777–1782 10.1002/jmv 21837795

[3] Hamprecht K, Maschmann J, Vochem M, Dietz K, Speer CP, et al (2001) Epidemiology of transmission of cytomegalovirus from mother to preterm infant by breastfeeding. Lancet 357: 513–518 10.1016/S0140-6736(00)04043-5 11229670

[4] Mussi-Pinhata MM, Pinto PCG, Yamamoto AY, Berencsi K, de Souza CBS, et al (2003) Placental transfer of naturally acquired, maternal cytomegalovirus antibodies in term and preterm neonates. J Med Virol 69: 232–239 10.1002/jmv.10271 12683413

[5] Nijman J, de Vries LS, Koopman-Esseboom C, Uiterwaal CSPM, van Loon AM, et al (2012) Postnatally acquired cytomegalovirus infection in preterm infants: a prospective study on risk factors and cranial ultrasound findings. Arch Dis Child Fetal Neonatal 97: F259–63 10.1136/archdischild-2011-300405 22247412

[6] Vollmer B, Seibold-Weiger K, Schmitz-Salue C, Hamprecht K, Goelz R, et al (2004) Postnatally acquired cytomegalovirus infection via breast milk: effects on hearing and development in preterm infants. Pediatr Infect Dis J 23: 322–327. 15071286 10.1097/00006454-200404000-00009

[7] Bevot A, Hamprecht K, Krägeloh-Mann I, Brosch S, Goelz R, et al (2011) Long-term outcome in preterm children with human cytomegalovirus infection transmitted via breast milk. Acta Paediatr 101: e167–72 10.1111/j.1651-2227.2011.02538.x 22111513

[8] Nijman J, van Zanten GA, de Waard AM, Koopman-Esseboom C, de Vries LS, et al (2012) Hearing in preterm infants with postnatally acquired cytomegalovirus infection. Pediatr Infect Dis J 31: 1082–1084 10.1097/INF.0b013e31825eb3e5 22592518

[9] Pignatelli S, Lazzarotto T, Gatto MR, Dal Monte P, Landini MP, et al (2010) Cytomegalovirus gN genotypes distribution among congenitally infected newborns and their relationship with symptoms at birth and sequelae. Clin Infect Dis 51: 33–41 10.1086/653423 20504230

[10] Lurain NS, Kapell KS, Huang DD, Short JA, Paintsil J, et al (1999) Human cytomegalovirus UL144 open reading frame: sequence hypervariability in low-passage clinical isolates. J Virol 73: 10040–10050. 10559318 10.1128/jvi.73.12.10040-10050.1999 PMC113055

[11] Stranska R, Schuurman R, Toet M, de Vries LS, Van Loon AM (2006) Application of UL144 molecular typing to determine epidemiology of cytomegalovirus infections in preterm infants. J Clin Microbiol 44: 1108–1110 10.1128/JCM.44.3.1108 16517906 PMC1393077

[12] Yan H, Koyano S, Inami Y, Yamamoto Y, Suzutani T, et al (2008) Genetic variations in the gB, UL144 and UL149 genes of human cytomegalovirus strains collected from congenitally and postnatally infected Japanese children. Arch Virol 153: 667–674 10.1007/s00705-008-0044-7 18273679

[13] Manuel O, Asberg A, Pang X, Rollag H, Emery VC, et al (2009) Impact of genetic polymorphisms in cytomegalovirus glycoprotein B on outcomes in solid-organ transplant recipients with cytomegalovirus disease. Clin Infect Dis 49: 1160–1166 10.1086/605633 19751151

[14] Murayama T, Takegoshi M, Tanuma J, Eizuru Y (2005) Analysis of human cytomegalovirus UL144 variability in low-passage clinical isolates in Japan. Intervirology 48: 201–206 10.1159/000081749 15812195

[15] Revello MG, Campanini G, Piralla A, Furione M, Percivalle E, et al (2008) Molecular epidemiology of primary human cytomegalovirus infection in pregnant women and their families. J Med Virol 80: 1415–1425 10.1002/jmv 18551604

[16] Arav-Boger R, Willoughby RE, Pass RF, Zong J-C, Jang W-J, et al (2002) Polymorphisms of the cytomegalovirus (CMV)-encoded tumor necrosis factor-alpha and beta-chemokine receptors in congenital CMV disease. J Infect Dis 186: 1057–1064 10.1086/344238 12355354

[17] Paradowska E, Studzińska M, Nowakowska D, Wilczyński J, Rycel M, et al (2012) Distribution of UL144, US28 and UL55 genotypes in Polish newborns with congenital cytomegalovirus infections. Eur J Clin Microbiol Infect Dis 31: 1335–1345 10.1007/s10096-011-1447-z 22048843

[18] Scanga L, Chaing S, Powell C, Aylsworth AS, Harrell LJ, et al (2006) Diagnosis of human congenital cytomegalovirus infection by amplification of viral DNA from dried blood spots on perinatal cards. J Mol Diagn 8: 240–245 10.2353/jmoldx.2006.050075 16645211 PMC1867599

[19] De Vries LS, Gunardi H, Barth PG, Bok LA, Verboon-Maciolek MA, et al (2004) The spectrum of cranial ultrasound and magnetic resonance imaging abnormalities in congenital cytomegalovirus infection. Neuropediatrics 35: 113–119 10.1055/s-2004-815833 15127310

[20] Chou SW, Dennison KM (1991) Analysis of interstrain variation in cytomegalovirus glycoprotein B sequences encoding neutralization-related epitopes. J Infect Dis 163: 1229–1234. 1709960 10.1093/infdis/163.6.1229

[21] Meyer-König U, Haberland M, von Laer D, Haller O, Hufert FT (1998) Intragenic variability of human cytomegalovirus glycoprotein B in clinical strains. J Infect Dis 177: 1162–1169. 9592998 10.1086/515262

[22] Shepp DH, Match ME, Lipson SM, Pergolizzi RG (1998) A fifth human cytomegalovirus glycoprotein B genotype. Res Virol 149: 109–114. 9602505 10.1016/s0923-2516(98)80086-1

[23] Chou SW (1990) Differentiation of cytomegalovirus strains by restriction analysis of DNA sequences amplified from clinical specimens. J Infect Dis 162: 738–742. 2167341 10.1093/infdis/162.3.738

[24] Yu ZS, Zou CC, Zheng JY, Zhao ZY (2006) Cytomegalovirus gB genotype and clinical features in Chinese infants with congenital infections. Intervirology 49: 281–285 10.1159/000093458 16714857

[25] Waters A, Hassan J, De Gascun C, Kissoon G, Knowles S, et al (2010) Human cytomegalovirus UL144 is associated with viremia and infant development sequelae in congenital infection. J Clin Microbiol 48: 3956–3962 10.1128/JCM.01133-10 20810771 PMC3020855

[26] Bale JF, Petheram SJ, Robertson M, Murph JR, Demmler G (2001) Human cytomegalovirus a sequence and UL144 variability in strains from infected children. J Med Virol 65: 90–96. 11505449

[27] Barbi M, Binda S, Caroppo S, Primache V, Didò P, et al (2001) CMV gB genotypes and outcome of vertical transmission: study on dried blood spots of congenitally infected babies. J Clin Virol 21: 75–79. 11255100 10.1016/s1386-6532(00)00188-8

[28] Picone O, Costa J-M, Leruez-Ville M, Ernault P, Olivi M, et al (2004) Cytomegalovirus (CMV) glycoprotein B genotype and CMV DNA load in the amniotic fluid of infected fetuses. Prenat Diag 24: 1001–1006 10.1002/pd.942 15614854

[29] Enders G, Daiminger A, Bäder U, Exler S, Enders M (2011) Intrauterine transmission and clinical outcome of 248 pregnancies with primary cytomegalovirus infection in relation to gestational age. J Clin Virol 52: 244–246 10.1016/j.jcv.2011.07.005 21820954

[30] Paradowska E, Jabłońska A, Studzińska M, Suski P, Kasztelewicz B, et al (2013) Distribution of cytomegalovirus gN variants and associated clinical sequelae in infants. J Clin Virol 58: 271–275 10.1016/j.jcv.2013.05.024 23806667

[31] Paradowska E, Jablonska A, Studzinska M, Kasztelewicz B, Zawilinska B, et al (2014) Cytomegalovirus glycoprotein H genotype distribution and the relationship with hearing loss in children. J Med Virol: 1–7. 10.1002/jmv 24615599

[32] Revello MG, Zavattoni M, Baldanti F, Sarasini A, Paolucci S, et al (1999) Diagnostic and prognostic value of human cytomegalovirus load and IgM antibody in blood of congenitally infected newborns. J Clin Virol 14: 57–66. 10548131 10.1016/s1386-6532(99)00016-5

[33] Boppana SB, Fowler KB, Pass RF, Rivera LB, Bradford RD, et al (2005) Congenital cytomegalovirus infection: association between virus burden in infancy and hearing loss. J Pediatr 146: 817–823 10.1016/j.jpeds.2005.01.059 15973325

[34] Ross S, Novak Z, Fowler KB, Arora N, Britt WJ, et al (2009) Cytomegalovirus blood viral load and hearing loss in young children with congenital infection. Pediatr Infect Dis J 28: 588–592 10.1097/INF.0b013e3181979a27 19478688 PMC3033214

[35] Nijman J, Van Loon AM, de Vries LS, Koopman-Esseboom C, Groenendaal F, et al (2012) Urine viral load and correlation with disease severity in infants with congenital or postnatal cytomegalovirus infection. J Clin Virol 54: 121–124. 22421537 10.1016/j.jcv.2012.02.017

[36] De Jong EP, Lopriore E, Vossen ACTM, Steggerda SJ, Te Pas AB, et al (2010) Is routine TORCH screening warranted in neonates with lenticulostriate vasculopathy? Neonatology 97: 274–278 10.1159/000255166 19887856

[37] Chang C-L, Chiu N-C, Ho C-S, Li S-T (2006) Frontal horn cysts in normal neonates. Brain Dev 28: 426–430 10.1016/j.braindev.2006.01.002 16503391 PMC7125929

[38] Leijser LM, Steggerda SJ, de Bruine FT, van Zuijlen A, van Steenis A, et al (2010) Lenticulostriate vasculopathy in very preterm infants. Arch Dis Child Fetal Neonatal Ed 95: F42–6 10.1136/adc.2009.161935 19457874

[39] Leruez-Ville M, Vauloup-Fellous C, Couderc S, Parat S, Castel C, et al (2011) Prospective identification of congenital cytomegalovirus infection in newborns using real-time polymerase chain reaction assays in dried blood spots. Clin Infect Dis 52: 575–581 10.1093/cid/ciq241 21292661

